# The educational background and qualifications of UK medical students from ethnic minorities

**DOI:** 10.1186/1472-6920-8-21

**Published:** 2008-04-16

**Authors:** IC McManus, Katherine Woolf, Jane Dacre

**Affiliations:** 1 Dept of Psychology, University College London, Gower Street, London WC1E 6BT, UK; 2 Academic Centre for Medical Education, Royal Free and University College Medical School, 4th Floor, Holborn Union Building, Whittington Campus, Highgate Hill, London N19 3LW, UK

## Abstract

**Background:**

UK medical students and doctors from ethnic minorities underperform in undergraduate and postgraduate examinations. Although it is assumed that white (W) and non-white (NW) students enter medical school with similar qualifications, neither the qualifications of NW students, nor their educational background have been looked at in detail. This study uses two large-scale databases to examine the educational attainment of W and NW students.

**Methods:**

Attainment at GCSE and A level, and selection for medical school in relation to ethnicity, were analysed in two separate databases. The 10^th ^cohort of the Youth Cohort Study provided data on 13,698 students taking GCSEs in 1999 in England and Wales, and their subsequent progression to A level. UCAS provided data for 1,484,650 applicants applying for admission to UK universities and colleges in 2003, 2004 and 2005, of whom 52,557 applied to medical school, and 23,443 were accepted.

**Results:**

NW students achieve lower grades at GCSE overall, although achievement at the highest grades was similar to that of W students. NW students have higher educational aspirations, being more likely to go on to take A levels, especially in science and particularly chemistry, despite relatively lower achievement at GCSE. As a result, NW students perform less well at A level than W students, and hence NW students applying to university also have lower A-level grades than W students, both generally, and for medical school applicants. NW medical school entrants have lower A level grades than W entrants, with an effect size of about -0.10.

**Conclusion:**

The effect size for the difference between white and non-white medical school entrants is about B0.10, which would mean that for a typical medical school examination there might be about 5 NW failures for each 4 W failures. However, this effect can only explain a portion of the overall effect size found in undergraduate and postgraduate examinations of about -0.32.

## Background

In the UK there is a concern that medical students and doctors from ethnic minorities underperform both in undergraduate and postgraduate examinations. In this paper we look firstly at a large population study of the educational achievement of all UK sixteen year-olds (the Youth Cohort Study), which reflects the pool from which all future UK medical students will be drawn, and we relate those results to a separate study based on data from UCAS (Universities and Colleges Admissions Service), looking at the educational qualifications of all university applicants, some of whom applied to medical school, and a proportion of whom were then accepted to study medicine. The primary interest throughout is whether, where, when and why ethnic minority students underperform relative to white students, and the extent to which any differences might explain the underperformance of medical students from ethnic minorities.

Medical students in UK universities are ethnically diverse, so that in recent years about 30% of home students (i.e. those resident in the UK, with UK nationality) come from ethnic minorities. That proportion has risen [1], surveys by one of us finding that about 10% of 1981 entrants were non-white, a figure which rose to 14% for those entering in 1986, and 22% for those entering in 1991 [2-5]. Official data from UCAS for 1996, 2001 and 2005, show that 30%, 33% and 30% of entrants for medicine and dentistry were non-white. (It should be noted that the statistics provided at the UCAS website [6] do not allow a separate analysis of medicine and dentistry in relation to ethnic group. Based on the datafile described below, the proportion of non-white entrants for medicine alone in 2005 was 31.2%, a value very similar to the UCAS figure for medicine 29.7% for medicine and dentistry combined. In the 2001 census, about 13% of those aged 15, 17% of those aged 16–19 and 19% of those aged 20–24 were from ethnic minorities, suggesting that ethnic minorities are somewhat over-represented amongst medical students compared with their proportion in the population as a whole.

In 1995, controversy erupted in the UK because of a higher failure rate in clinical examinations of non-white students in the University of Manchester [7,8]. As a result of that controversy, a re-analysis of data from the 1981 and 1986 cohorts found that amongst students taking University of London final examinations, the non-white students performed less well overall than did white students, the difference being found in all types of examination (multiple-choice questions, essay, oral and clinical), and in all the five main subjects (Medicine, Surgery, Obstetrics and Gynaecology, Pathology and Pharmacology) [9]. The reasons for the differences were not clear, and could not be explained away in terms of a range of background measures, be they demographic, educational or psychometric.

In the past ten years, further data have accumulated on the relatively poorer performance of UK non-white medical students and doctors in undergraduate and postgraduate examinations, non-whites performing less well in undergraduate clinical examinations [9-14], as well as in the postgraduate examinations of the Royal Colleges of Physicians (the MRCP(UK) [15,16]), and the Royal College of General Practitioners (MRCGP [17,18]).

The published studies comparing the undergraduate and postgraduate performance of white and non-white medical students and doctors can be compared by calculating *d*, a standard meta-analytic measure of effect size techniques (see Method, below, for details). Effect sizes are a standard statistical method for comparing disparate results across studies, by converting all results to a standard scale similar to *z *scores. When there are two groups, then the effect size is a measure of the distance apart of the two means measured in standard deviation units. A conventional interpretation of effect sizes, due to Cohen, is that effect sizes of .2, .5 and .8 can be described as small, medium and large [19]. Considering the published studies described above, the effect sizes were -.097 and -.283 [9], -.237, -.291, -.580 and -.273 [10], -.665 [12], -.579 [13], -.492 [14], and -.277, -.344 and -.391 [15]. We are currently preparing a formal meta-analysis of these and other unpublished data, but for present purposes it suffices to say that the mean effect size found in these studies is -.375, with a median of -.318. Viewing the data conservatively, for the present paper we will use the median effect size of -.32 as typical of published studies. Using Cohen's terminology, it is between medium and small.

Although the problem of the underperformance of ethnic minorities in medical education has been much discussed, the underperformance is not unique to medical studies, Richardson in a recent analysis of HESA (Higher Education Statistics Authority) data for graduates of UK degree-giving bodies in 2005 [20], found a consistent under-performance of non-white students, which for the cohort aged 21–24 at graduation gave an odds ratio comparing the likelihood of NW students with W students gaining > good degrees = (i.e. I or II.i) of 0.518, equivalent to an effect size of B0.363, similar to the averaged effect reported in medicine. Similar results have also been reported by Connor et al [21], who have also emphasised that the participation rate of ethnic minorities in higher education is higher than for the white population, although there are differences between the various groups. Neither is underperformance of ethnic minorities restricted to university performance. Kirkup et al [22], in a study for the Sutton Trust, with what was probably not an entirely representative sample of UK students taking A-levels, found differences between white and non-white students, with estimated effect sizes of -0.159 for GCSEs, -0.239 for A-level grades, and -.514, -.141, -.571, -.588 and -.199 for the Critical reading, Mathematics, Writing, Writing-Multiple Choice, and Writing: essay assessments of the SAT test (giving a mean effect size of -.403 for the five SAT components); overall the seven measures had a mean effect size of -.345. The summary data from the Research Study of the Department for Education and Skills (DfES) [23] for pupils taking assessments in 2005 allow the calculation of effect sizes for differences between white and non-white pupils of -.215 at Key Stage 1 (age 7), -.211 at Key Stage 2 (age 11), -.178 at Key Stage 3 (age 14), and -.028 at Key Stage 4 (GCSEs) (effect sizes being calculated separately from the percentages of pupils attaining expected levels of achievement in the three separate components of each Key Stage, using the data provided in table 8 of the Research Report [[23], p.42], with all non-white pupils (excluding 'Unclassified') being combined together, and weighted by the Ns provided for the data at the (former) DfES website [24]) In pre-school children, the second sweep of the Millennium Cohort Study of a large, representative group of UK three-year olds, found lower performance in ethnic minorities on two cognitive scales, with an effect size of -.28 for the Naming Vocabulary scale of the British Ability Scales, and an effect size of -.11 for the Bracken Basic School Readiness Scale [25].

A range of explanations have been put forward for the underperformance of ethnic minorities in medical school, often revolving around discrimination of some form or another (e.g. Wass *et al *[12]), although such explanations have problems in explaining underperformance in computer-marked multiple-choice examinations (e.g. at the undergraduate level in [12] and at postgraduate level in Part 1 and Part 2 of the MRCP(UK) [15]). A detailed analysis of a large number of candidates taking PACES, the MRCP(UK) clinical examination, also finds that there is little association between the performance of candidates in relation to their own ethnicity and that of their examiners [15].

There are relatively few consistent predictors of educational outcome at university level and beyond, although one measure which does continue to make a statistical prediction, is A level examination results, the > Advanced level = examination taken by would-be university entrants in the UK, particularly in England, Wales and Northern Ireland (most applicants in Scotland taking Highers). A level results have been shown to predict performance at university, both in general [26,27] and in medicine for both undergraduate [14,28,29] and post-graduate medical examinations [28,29]. If there were differences in performance of non-white entrants at A level examinations, then that might in part explain some of the differences found in performance of ethnic minorities at medical school.

In the UK, school education is compulsory until the age of 16, when the GCSE (General Certificate of Secondary Education) exams are taken by almost all students in schools, the only exception being a small minority with severe educational, intellectual or behavioural problems (and for a detailed study of them and their characteristics see Cassen & Kingdom [30]). At the age of 16, students can leave education, and many choose to do so. Those who do stay on, either at school (Sixth Form), or at colleges of further education, can take a range of qualifications, of which the main ones relevant to the question of medical school applications, are the A level examinations (or Highers in Scotland), with only a few medical school applicants taking instead the International Baccalaureate or the Welsh Baccalaureate, which is currently being piloted).

Education after the age of sixteen is often called post-compulsory education, with the important implication that students *choose *to stay on, and also, in staying on, they *choose *which subjects to study and for what purposes. The element of choice in post-compulsory education complicates all statistical analyses of differences between groups, ethnic or otherwise, because one is not dealing with an entire population sample, but instead a sample that is self-selected, having chosen to be doing what it is doing, and which therefore, in statistical terms, is potentially biased. Any differences between groups may therefore result either from differences in true ability or from differences in the choices that have been made, for whatsoever reason.

In this paper we wish to look carefully at academic qualifications in relation to ethnic origin, initially in the Youth Cohort Study (YCS), a representative, population sample of 16-year olds in England and Wales, in whom performance at GCSE is well-described, as also are many aspects of performance in post-compulsory education. Our principle interest will be in factors relevant to the pool of potential medical students, and we note that more general aspects of the attainment gap in minority ethnic pupils in school have been reviewed comprehensively elsewhere [23,31]. Because medical students are a relatively small proportion of the total population, we will also compare the analysis of the representative sample of students from YCS with a parallel analysis of a separate database of all UK applicants to university, who have applied through UCAS (Universities and Colleges Admissions Service). The data in the latter are of course far less comprehensive in relation to the population as a whole, because many of the population do not apply to university, but they allow a analysis of applicants and acceptances to a range of university subjects, and in particular to medicine. A joint analysis of the YCS data and the UCAS data allows a more complete picture to appear.

## Methods

### i) Youth Cohort Study (YCS)

The YCS has been carried out annually or biennially since 1985, and the current analysis is for Cohort 10, who were aged 16 or 17 when the first sweep of the survey was carried out in Spring 2000, mostly having taken GCSEs the summer before, in May 1999 [32,33]. Further sweeps were carried out in November 2000 (Sweep 2), and March 2002 (Sweep 3), the latter being of particular importance as A level results are available. The initial sample consisted of a representative sample of 25,000 pupils from all schools in England and Wales (excluding special schools and those with fewer than 20 pupils) who were of school-leaving age during the academic year 1998–1999, and reached the age of 16 by Aug 31^st ^1999. Questionnaires for sweeps 2 and 3 were only sent to those replying to previous sweeps. Details of the survey are available in the survey codebook [33], which includes details of all three sweeps. The YCS provides weighting variables to take into account any biasses in responding in the surveys, and these have been applied in all analyses described here, so all values are representative of population values.

### ii) UCAS dataset

Data were provided by UCAS for all applicants applying for admission to university in the autumn of 2003, 2004 and 2005. Information was available on the university subject for which the applicant had applied, as well as specific information on whether they had applied for medicine, and whether they had been accepted for medicine. Demographic information was also available on age, sex, ethnicity, place of residence, parental occupation, and parental education, and the educational data included the subject and grade of all A levels and AS levels taken, as well as the standard UCAS tariff score (see below).

Statistical analysis used SPSS 11.5 for most analyses, with LISREL 8.54 being used for structural equation modelling. Effect sizes for continuous variables are calculated as *d *= (mean _W_-mean _NW_)/SD _W_. Odds ratios, calculated as *OR *= *p*_*W *_× (1-*p*_*NW*_)/(*p*_*NW *_× (1-*p*_*W*_)), are converted to effect sizes using the method of Chinn [34]. Negative effect sizes should be interpreted as NW students performing less well or having a lower mean than W, or NW having a lower proportion or probability of an event than W.

## Results

### Definition of ethnicity

The definition of ethnicity is complex, with different studies using different criteria and classifications. It is also probable that there are differences between varying groups of ethnic minorities. However for our present purpose we are primarily interested in those studies which, for convenience and other reasons, simply compare > white = (W) students with > non-white = (NW) students. In the medical school population the majority of non-white students are from the Indian sub-continent (primarily Indian, Pakistani, Bangladeshi and other), although there are students from other groups. Supplementary table 1 [see Additional file 1] provides a complete description of all individuals using the classificatory schemes of YCS and UCAS. Although so doing inevitably simplifies a complex process, for ease of explication, we will divide students into just two groups, in order to be able to see the bigger picture more straightforwardly. We acknowledge in advance that many further, more detailed, analyses could be carried out.

### The Youth Cohort Study

#### Performance at GCSE

**Figure 1:**
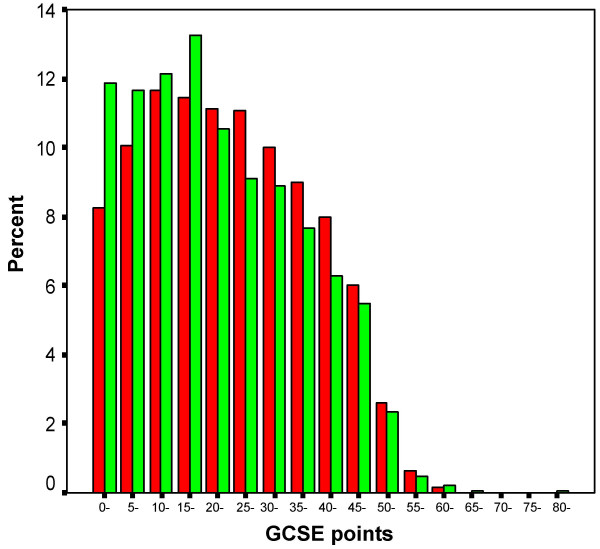
The distribution of GCSE points gained by W and NW students, shown in red and green respectively. GCSE points are group as 0–4, 5–9, 10–14, etc..

Almost all students took GCSEs, only 649/13698 (4.7%) not taking any GCSEs, and those individuals were excluded from further analysis. The simplest overall measure of GCSE performance is the total number of points attained (where A = 5, B = 4, C = 3, D = 2, E = 1, else = 0). It should be noted that although for a number of years GCSEs have awarded a grade of A*, which is higher than A, those A* results are unfortunately not distinguished from A in the YCS database. Inevitably any simple points total is influenced by the total number of GCSE examinations for which a student chooses to enter or is allowed to enter, and so we also provide the mean grade attained at GCSE. Overall there is only a very small absolute difference in the number of GCSEs taken by white and non-white candidates, which just reaches significance (see Table 1), but there is a highly significant difference in the number of points and the mean grade attained, p < .001 in each case. Figure 1 shows the distribution of points for W and NW students, and although it is clear that the NW students have a lower mean, which can be seen particularly in the left-hand tail, at the top end of the distribution the differences are far less marked, such that the number of A grades attained is more similar in W and NW students, and the proportion of students gaining 6 or more A grades B the pool of those with a realistic chance of subsequently applying for medical school B is not significantly different in the W and NW candidates, 10.7% and 9.8% respectively, the effect size being B.054. Table 1 also shows the proportions gaining A grades in the main subjects taken, and for most subjects there is no significant difference between W and NW candidates.

**Table 1:** GCSE achievement of W and NW students (YCS).

	White Mean (SD) N = 11273	Non-White Mean (SD) N = 1776	Significance	Effect size
Number of GCSEs taken	8.65 (1.90)	8.55 (1.99)	t = 2.07, p = .039	d = -.053
Total number of GCSE points	24.12 (14.18)	22.01 (14.44)	t = 5.81, p < .001	d = -.148
Mean GCSE points	2.65 (1.34)	2.44 (1.38)	t = 5.89, p < .001	d = -.156
Number of GCSEs at A grade	1.44 (2.62)	1.32 (2.54)	t = 1.80, p = .072	d = -.046
Average difficulty of GCSEs taken	.773 (.029))	.771 (.029)	t = 1.93, p = .054	d = -.048
	White N (%)	Non-White N(%)	Chi-squared (p)	
Percent with 6 or more GCSEs at grade A	1206/11272 = 10.7%	174/1602 = 9.8%	χ^2 ^= 1.32, 1df, p = .251	OR = 0.907 (CI .77 – 1.07) d = -.054

*Percent with A grade in particular subjects:*				

English language	1580/10798 = 14.6%	216/1666 = 13.0%	.190 (1) p = .190	-
English Literature	1561/9272 = 16.8%	196/1391 = 14.1%	6.62 (1) p = .010	-
Maths	1321/9485 = 13.9%	208/1168 = 15.1%	1.41 (1) p = .236	-
French	1219/5798 = 21.0%	151/783 = 19.3%	1.27 (1) p = .260	-
Geography	969/4678 = 20.7%	110/513 = 17.7%	3.17 (1) p = .075	-
History	916/3647 = 25.1%	122/369 = 24.8%	.017 (1) p = .897	-
Art	799/3427 = 23.3%	123/560 = 22.0%	.494 (1) p = .482	-
Craft	869/4508 = 16.2%	101/828 = 12.2%	8.54 (1) p = .003	-
German	521/2508 = 20.8%	55/303 = 18.2%	1.14 (1) p = .286	-
Business Studies	251/1910 = 13.1%	50/346 = 14.5%	.43 (1) .510	-
Double Science	1141/8125 = 14.0%	158/1182 = 13.4%	.39 (1) p = .531	-

#### Difficulty of GCSEs

Although almost all students take GCSE at age 16, there is an element of choice in the particular subjects chosen for study. There is also good evidence that not all subjects are equally difficult, some such as Chemistry, Physics, Biology and Latin being harder subjects in which to gain top grades than Art, Drama, and Sociology [35]. We therefore calculated a measure of the average difficulty of all the GCSE subjects that had been taken by a student, based on Coe = s estimates of the Rasch difficulty for an A* grade. The choice of A* for this calculation has little impact overall, since the Rasch difficulties for different grades are, to a large extent, parallel [35], and A* has the advantage for present purposes that it is the grade which most medical students would be expected to achieve. Table 1 shows that there is no significant difference between W and NW students, meaning that NW students take GCSEs of similar difficulty to W students.

#### Demographic factors

W and NW students differ in a number of demographic factors, as can be seen in Table 2, the NW students coming from a somewhat lower socio-economic group, and their parents having somewhat less education, although interestingly there are no differences in the proportions attending private schools.

**Table 2:** Demographic measures for W and NW students (YCS).

	White N = 11273	Non-White N = 1776	Significance
*Sex*			
Male	5657 (50.2%)	898 (50.6%)	χ^2 ^= .089 (1) p = .765
Female	5616 (49.8%)	878 (49.4%)	
*Socio-economic group*			
V (Unskilled)	482 (4.7%)	70 (5.6%)	χ^2 ^= 25.71 (4) p < .001
IV (Semi-skilled)	1181 (11.5%)	188 (15.1%)	
III (Skilled manual)	3940 (38.3%)	495 (39.7%)	
II (Other non-manual)	2218 (21.5%)	251 (20.1%)	
I (Managerial/profssional)	2477 (24.1%)	243 (19.5%)	
*Parental education*			
Pre-A-level	6935 (61.5%)	1261 (71.0%)	χ^2 ^= 75.72 (2) p < .001
A levels	1790 (15.9%)	159 (9.0%)	
Degree	2547 (22.6%)	356 (20.0%)	
*School type*			
State	10488 (93.0%)	1652 (93.0%)	χ^2 ^= .015 (1) p = .093
Private	784 (7.0%)	125 (7.0%)	

#### Correlation and regression analyses

Table 3 shows simple Pearson correlations of the measures of GCSE achievement with background variables. As in most complex social data there are correlations between very many of the measures. Of particular interest are that non-white ethnicity correlates with taking slightly fewer GCSEs and gaining fewer points at GCSE, as well as coming from a lower socio-economic background and having less parental education. However it is also the case that non-white ethnicity does not correlate with attending a private school, taking more difficult GCSEs, or being female. There are also many significant correlations between background variables, such as between social class and all of the other measures. A multiple regression of GCSE points on the seven background factors found that all were significant at p < .001, higher GCSE points being predicted by taking more GCSEs (β = .604), greater level of parental education (β = .152), attending a private school (β = .147), taking *more *difficult GCSEs (β = .116), coming from a higher socio-economic group (β = .110), being female (β = .081), and being white (β = .026), the variables together accounting for 55% of the variance, of which 43% was accounted for by number of GCSEs taken. A similar analysis using logistic regression and with the attainment of 6 or more A grades as the dependent variable, found highly significant effects of all the same variables at p < .001, with the important exception that ethnicity was *not *significant. If ethnicity was forced into the logistic regression it had a significance level of .437.

**Table 3:** Pearson correlations between achievement at GCSE and background variables. Weighted N for cells varies between 11,544 and 13,049. Significance levels are shown as: *** p < .001; ** p < .01; * p < .05.

	Total points at GCSE	Number of GCSEs taken	Difficulty of GCSEs taken	Independent (private)	Female sex	Higher socio- economic	Greater parental	Non-white ethnicity
Total points at GCSE	1.000	0.658 ***	0.187 ***	0.265 ***	0.115 ***	0.295 ***	0.338 ***	-0.051 ***
Number of GCSEs taken	0.658 ***	1.000	-0.006	0.057 ***	0.058 ***	0.159 ***	0.167 ***	-0.018 *
Difficulty of GCSEs taken	0.187 ***	-0.006	1.000	0.286 ***	0.000	0.098 ***	0.140 ***	-0.017
Independent (private)	0.265 ***	0.057 ***	0.286 ***	1.000	0.002	0.153 ***	0.228 ***	0.001
Female sex	0.115 ***	0.058 ***	0.000	0.002	1.000	-0.025 **	0.004	-0.003
Higher socio- economic group^+^	0.295 ***	0.159 ***	0.098 ***	0.153 ***	-0.025 **	1.000	0.318 ***	-0.044 ***
Greater parental education	0.338 ***	0.167 ***	0.140 ***	0.228 ***	0.004	0.318 ***	1.000	-0.050 ***
Non-white Ethnicity	-0.051 ***	-0.018 *	-0.017	0.001	-0.003	-0.044 ***	-0.050 ***	1.000

^+ ^Note: For convenience of interpretation, socio-economic group here and in other analyses has been rescored so that *higher *groups have *higher *scores (i.e. I = 5, II = 4, III = 3, IV = 2 and V = 1).

#### Path analysis

**Figure 2:**
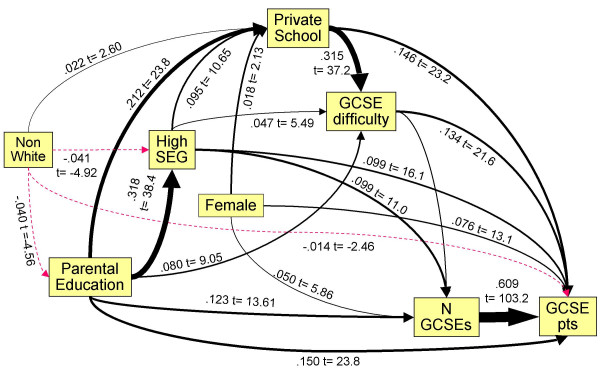
Path model of direct and indirect influences of background factors upon GCSE performance. Path coefficients and t-statistics are shown alongside paths, the thickness of paths is proportional to the path coefficient, and negative paths are shown as red, dashed lines.

Achievement at GCSE depends on a number of variables, and as Table 3 shows, those variables are themselves inter-related, both in correlational and causal terms. We therefore used LISREL to fit a path model, shown in Figure 2. The causal ordering was chosen on the basis that the number of GCSEs taken and the difficulties of the GCSEs taken were both immediately prior to the total points attained, and that type of schooling was prior to taking GCSEs. The sex of the student, being fixed throughout the student = s life, was prior to all educational variables. The remaining variables were related to the student = s families as much as to the student themselves, and hence were earlier in causal terms, with parental socio-economic group being likely to be secondary to parental education, and parental ethnicity prior to parental education. The logic is that the ethnicity of parents and offspring is generally the same, and fixed throughout the lifespan of parents and offspring, whereas education of both parents and offspring is less fixed, so that in any causal model, the ethnicity of individuals and their parents is to the left of measures of education. The number of individuals of mixed ethnicity (who are here classified as NW) is relatively small, and hence little affects the above argument, and it is still the case that an individual's ethnicity precedes their education causally.

The model was fitted by firstly using a saturated model in which all variables to the left of a variable could cause all variables to the right of the variable, through the BETA matrix in LSIREL. Paths which were non-significant at the 0.05 level were dropped sequentially from the model, with the least significant first, until all paths remaining were significant.

The final fitted model is shown in Figure 2. The overall goodness of fit was excellent, with χ^2 ^= 8.92, 8 df, p = .349, a goodness of fit index of 1.000, and an adjusted goodness of fit of 0.999. Figure 2 shows a number of readily interpretable effects. Higher parental education results in a higher parental socio-economic group, and both factors result in a child being more likely to take part in private schooling. Private schools are more likely to put children in for more difficult GCSEs, and the children also gain more points at GCSE. Total GCSE points is particularly dependent on number of GCSEs taken, and the difficulty of the GCSEs taken, and there are also direct influences of private schooling, as well as effects of sex, parental social class, and parental education which are not explained by schooling. Finally it should be noticed that ethnicity has multiple effects, as a result of NW students having less parental education and coming from a lower socio-economic group, but after taking those into account, ethnic minorities are somewhat *more *likely to attend private schools.

To summarise the results so far, NW students do achieve slightly less well at GCSE, but that is largely, but not entirely, mediated by a number of background variables such as parental education and type of schooling. In particular, the numbers of NW students attaining good grades at GCSE (6 or more As) is similar to that in W students, both in a simple analysis, and also after taking background factors into account. From the point of view of understanding the potential pool of medical school applicants B those performing particularly well at GCSE, in other words B W and NW students do not show significant differences in GCSE achievement.

### Choosing to take A levels

A levels are taken as a part of post-compulsory education in the UK, so that students firstly must choose to take A levels, and then they must choose which particular A level subjects to take. Medical schools typically require science subjects to have been studied, at least in part, and a majority of schools require a good grade in A level Chemistry. A levels undoubtedly differ in their relative difficulty, and Chemistry is widely perceived as more difficult than most other subjects, a perception which it would seem is correct [36]. To enter the pool of potential medical students a student must therefore in most cases take A level sciences in general, and A level Chemistry in particular.

**Figure 3:**
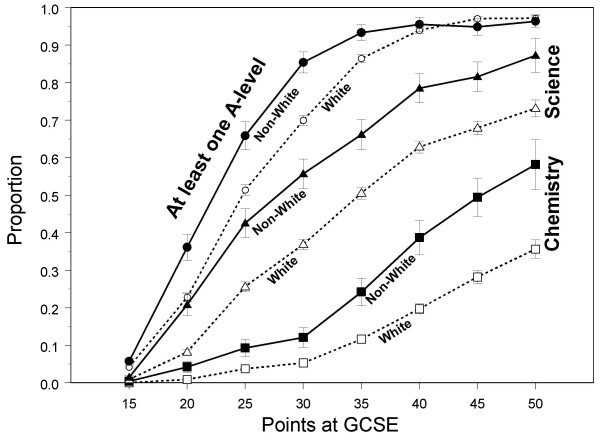
Proportion of W (Open points and solid lines) and NW students (solid points and dashed lines) taking at least one A-level (circles), at least one science A-level (triangles), and chemistry A-level (squares), in relation to points gained at GCSE. Error bars show ± one standard error.

YCS asked students in Sweep 1, who were surveyed about six months after getting their GCSE results, whether they were continuing in education, and in particular whether they were taking A levels, and if so, in what subjects. We have looked at three simple summary statistics; whether a student was taking one or more A levels (excluding General Studies), whether a student was taking one or more science A levels, and whether a student was taking Chemistry at A level. Table 4 shows that although a similar proportion of NW and W students go on to take A levels (despite NW students having somewhat lower GCSE grades overall), NW students are significantly *more *likely to take science A levels, and are nearly twice as likely to take A level chemistry in particular, one in nine of all NW students in the population taking A level chemistry in comparison with one in fifteen of all W students in the same age cohort. The fact that an equal proportion of W and NW students go on to take A levels is surprising, given the somewhat lower achievement of NW students at GCSE. Figure 3 shows the proportion of students going on to A levels in relation to GCSE points, and it is clear that at almost all levels of achievement, NW students are more likely to take A levels than are W students. Logistic regression confirms, after taking GCSE points into account, that non-white students are 1.74 times (95% CI 1.48–2.04; effect size = .306) more likely to take at least one A level, 2.08 times (95% CI 1.80–2.42; effect size = .405) more likely to take at least one science A level, and 2.61 times (95% CI 2.15 – 3.16; effect size = .530) more likely to take chemistry A level. In so far as GCSEs are predictive of A level achievement, as will be shown below, it seems likely therefore that NW students will perform less well at A level than W students.

**Table 4:** A-levels in W and NW students (YCS).

	White Mean (SD) N	Non-White Mean (SD) N	Significance	Effect size
Total number of points achieved at A level	17.71 (9.17) N = 2376	16.42 (9.15) N = 355	t = 2.474 p = .013	-.141
	White N (%)	Non-White N(%)		Odds Ratio (95% CI)
Taking one or more A-levels (excluding General Studies)	4666/11273 = 41.4%	738/1777 = 41.5%	χ^2 ^= .012 (1) p = .911	OR = 1.01 (.91–1.11) d = .005
Taking one or more science A-levels	2701/11273 = 24.0%	515/1777 = 29.0%	χ^2 ^= 20.84 (1) p < .001	1.30 (1.16–1.45) d = .145
Taking A-level Chemistry	744/11273 = 6.6%	202/1776 = 11.4%	χ^2 ^= 51.98 (1) p < .001	1.82 (1.54–2.14) d = .331

#### Regression analysis and path analysis

**Figure 4:**
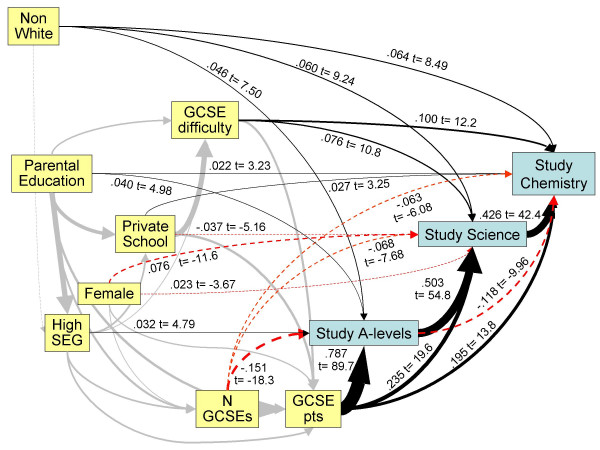
Path model of influences of background factors and GCSE performance (variables in yellow) upon taking of A-levels, science A-levels and A-level chemistry (variables in blue). Path coefficients and t-statistics are shown alongside paths, the thickness of paths is proportional to the path coefficient, and negative paths are shown as red, dashed lines. Paths previously shown in figure 2 are in pale grey without coefficients, to avoid undue confusion.

Ethnic differences in aspirations to take A levels were explored further using multiple regression and structural equation modelling, looking at taking of A-levels in relation to the eight variables shown in Figure 2 that relate to GCSE attainment and background characteristics. Forward entry logistic regression with taking of one or more A levels as the dependent variable, and the eight background variables as predictors, found significant effects of all the measures except private schooling. Predictors of taking A levels, in order of entry into the regression (but with significance levels calculated after taking all other variables into account) were higher numbers of GCSE points (p < .001), taking fewer GCSEs (p < .001), being non-white (p < .001), coming from a higher socio-economic group (p < .001), being male (p < .001), having greater parental education (p < .001), and taking more difficult GCSEs (p = .027). NW students were 1.979 times more likely to take A levels than white students (effect size = .377), all other background factors being taken into account. Very similar results were obtained for taking at least one science A level, and for taking Chemistry A level, NW students being 2.370 times more likely to take a science A level (effect size = .477), and 2.735 times more likely to take Chemistry A level (effect size = .556). Figure 4 shows a path model for taking A levels, and not only can the effects of ethnicity be seen, but it is also clear that NW students are more likely to take a science A level, even after taking into account the likelihood of taking A levels, and are more likely to take Chemistry A level, even after taking into account the fact that they are taking science A levels.

Although overall NW students have poorer achievement at GCSE, a previous analysis here had suggested that NW students are equally likely as W students to achieve very high GCSE grades (6 or more A grades or better). The logistic regressions described in the previous section were therefore repeated only for students with 6 or more A grades at GCSE. Overall there was now no effect of ethnicity on taking one or more A-levels, although that largely reflects the fact, seen at the right hand end of Figure 3, that 97% of such a high achieving group go on to take A levels. However, a similar analysis for the taking of science at A level and the taking of Chemistry showed that even among GCSE high-achievers, NW students were 2.85 times more likely to take a science A level (p < .001; effect size = .579), and 2.45 times more likely to take Chemistry A level (p < .001; effect size = .495).

#### A level difficulty

As with GCSEs, not all A level subjects are equally difficult. We therefore used the data of the CEM Centre at the University of Durham [36] to calculate a difficulty score for the subjects being taken by each student at A level. Multiple regression of A level difficulty on the six background variables and two measures of GCSE attainment shown in Figure 2 found that more difficult A levels were taken by students with more GCSE points (p < .001), but who had taken more difficult (P < .001) but somewhat fewer GCSEs (P < .001), were female (p < .001), and were non-white (p < .001). However, since Chemistry is one of the most difficult of A levels, the latter effect is perhaps not unexpected. Repeating the analysis but including a variable indicating whether or not students were studying chemistry at A level meant that the effect of ethnicity was no longer significant (p = .207).

In summary, despite having somewhat poorer overall attainment at GCSE than W students, NW students are more likely to go on to take A levels, and in particular science A levels and Chemistry A level, which tend to be more difficult. The same was also true of science A levels and Chemistry A level amongst the high achieving students with 6 or more A grades at GCSE.

### Attainment at A level

YCS was carried out in several sweeps, with information on GCSE results and intentions to take A level obtained at sweep 1. Information on actual attainment at A level was obtained only at sweep 3, and inevitably the response rate was much poorer. Of the original 25,000 individuals in the sampling frame, 13,699 responded to sweep 1, and only these individuals were sent sweep 2, of whom 10,100 responded. Only these 10,100 individuals were sent sweep 3, of whom 7,971 responded to sweep 3 (and of course many of these students did not take A levels). Nevertheless, because the sampling frame is well characterised, YCS provides appropriate weighting factors to take response biases into account, and the weighting has been applied in the following analyses, so that estimates are likely to be applicable to the population as a whole. Information at sweep 3 is less comprehensive, and little apart from examination achievement is directly relevant to the study of medicine as such.

#### A level grades in relation to GCSE grades

**Figure 5:**
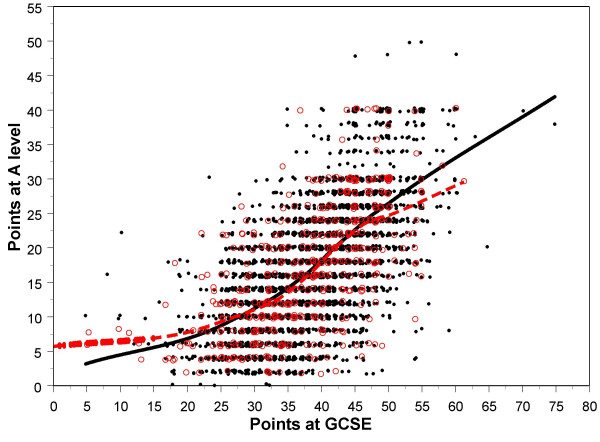
The relationship between points achieved at GCSE and points achieved at A-level, separately for W students (solid black points and solid black line) and NW students (open red points and dashed red line). All data points have been jittered slightly so that they do not overlap. The fitted lines are loess regression lines.

For present purposes we are only interested in A levels, and not AS levels, since most medical students have at least three A levels, and they are the main criterion for medical student selection. As is also the case in many medical schools, we have also not included General Studies as a > proper = A level subject. Figure 5 shows a scatter plot of total points at A level (calculated as was conventionally done by UCAS as A = 10, B = 8, C = 6, D = 4, E = 2, otherwise 0), in relation to total points at GCSE, with a lowess line fitted separately for W and NW students. The overall correlation is 0.628 (N = 2456), and was 0.633 (N = 2131) in the W students, and 0.606 (N = 326) in the NW students. It is also clear, in Figure 5, that the fitted lowess regression lines are very similar. To a first approximation therefore, GCSE achievement predicts A level outcome equally well in W and NW students, although there is a trend towards NW students with high GCSE grades underachieving to some extent at A level. Regression of A level points on GCSE points showed that the linear component of GCSE points accounted for 39.5% of the variance in A level points. Inclusion of ethnicity did not significantly improve the fit of the model (R^2 ^change < 0.1%; p = .470), although inclusion of an ethnicity *x *GCSE points interaction was significant (F(1,2472) = 12.7, p < .001) accounting for a further 0.3% of the variance. Fitting lines separately to W and NW groups, showed that the unstandardised regression coefficient was somewhat higher in W students (b = .660, SE = .017; beta = .633, R^2 ^= 40.1%) than in non-White students (b = .515, SE = .038, beta = .606, R^2 ^= 36.7%). GCSE grades therefore predict A levels slightly better in W than NW students, although the effect is small, and the broad picture is of similarity. Since NW students taking A levels have lower GCSE achievement than W students, it can be predicted that NW students will perform less well at A level than will W students.

#### Actual taking of A levels

Of the 13,049 individuals in sweep 1 of YCS, only 6692 (51.3%) responded on sweep 3 when A level results were asked for, and of these only 2731 had actually taken one or more A levels. (Note that these figures are slightly different from those cited at the beginning of the section on A level achievement as those figures are raw counts of number of respondents, whereas the figures quoted here, and subsequently, refer to weighted responses). Of the 3478 individuals at sweep 1 who said they did not intend to take A levels, only 74 (2.1%) actually did, with no difference in proportion between W and NW (2.1% and 2.4% respectively). However of the 3214 individuals at sweep 1 who said they intended to take A levels, 2657 (82.7%) actually did, and the proportion was higher in W students (2314/2780 = 83.2%) than in NW students (342/433 = 79.0%), a significant difference (chi-squared = 4.73, 1df, p = .030; odds ratio = 0.760, effect size = B.152), suggesting that more NW than W students had decided their aspirations were inappropriate or impractical, although the data cannot decide between those possibilities. Those not actually taking A levels who had said they would, had significantly lower GCSE point scores (mean = 29.1, SD = 10.24, N = 557) than those who did take A levels as intended (mean = 48.56, SD = 8.68, N = 2657, t = 22.76, p < .001). Similarly, although fewer NW students actually took A levels than said they would, the NW students who actually took A levels had significantly lower GCSE points than the W students (W: mean = 38.62, SD 8.63, N = 2376; NW: mean = 36.32, SD = 10.39, N = 355; t = 4.56, p < .001).

#### A level achievement

**Figure 6:**
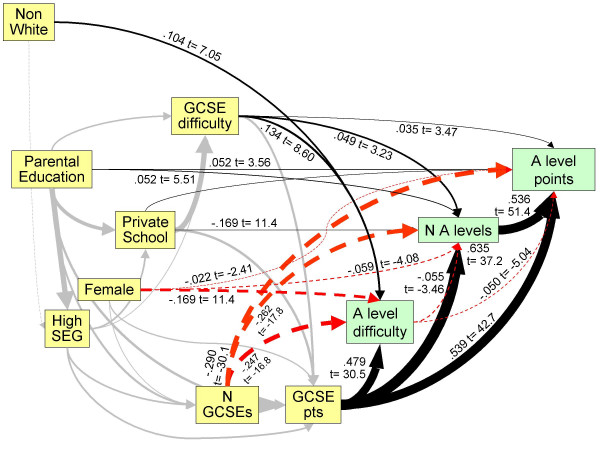
Path model of influences of background factors and GCSE performance (variables in yellow), upon A-level difficulty, number of A levels taken, and A-level performance (variables in pale green). Path coefficients and t-statistics are shown alongside paths, the thickness of paths is proportional to the path coefficient, and negative paths are shown as red, dashed lines. Paths previously shown in figure 2 are in pale grey without coefficients, to avoid undue confusion.

Given the lower achievement at GCSE it is not surprising that NW students also had lower total point scores at A level (see Table 4). Numbers taking chemistry and other individual subjects were too small to make useful comparisons. The effect just described is a simple effect, without taking background factors into account. As before it is useful to summarise the data on the different variables determining A level performance by means of a path analysis, and Figure 6 shows a LISREL model for the determination of A level points in terms of nine background variables. The most important determinant of A level points is the number of A levels taken, for fairly trivial reasons. GCSE points also determine A level points, both directly, and also indirectly via the number of A levels taken, better students at GCSE taking more A levels. Better students at GCSE also tend to take slightly more difficult A level subjects, and as a result do slightly less well than if they had taken easier subjects. Students taking more difficult subjects at GCSE also tend to take more difficult subjects at A level. Schooling, socio-economic group, and ethnicity do not have direct effects upon A level performance, although they have a range of indirect effects, whereas parental education level has direct effects, on both GCSE points and on A level points. Of particular interest is that there is no direct effect of ethnicity upon A-level performance, all effects being mediated indirectly via background factors and measures of performance at GCSE.

#### The UCAS dataset on applicants and entrants to universities

The YCS is a useful database for looking at the entire population of individuals taking GCSEs and then A levels. However, the numbers involved are too small to be useful for looking at applicants and entrants for particular university subjects. The UCAS database is extremely large, the three years from 2003–2005 having a total of 1,484,650 applicants, 476,467 in 2003, 486,028 in 2004 and 522,155 in 2005, of whom 52,557 applied for medicine, and 23,443 were accepted at medical school. However the database is weak in terms of the richness of the background variables, having only A levels, and not GCSEs, for instance, and having only a limited number of demographic variables. And of course, of necessity, it can only look at those individuals who have applied to university. Nevertheless, the UCAS data complements and supports the YCS data.

#### A level scores

Although most of UCAS = published analyses are in terms of its > tariff score = [37], the tariff score has several disadvantages when thinking primarily about medical school applicants. The tariff combines different qualifications which are on different metrics, but that has the disadvantage that the origin of any particular tariff is far from clear. For instance, a candidate gaining B grades at three A levels will receive 3 @ 100 = 300 points, as also will a candidate gaining two As and a D (120+120+60), as also will a candidate taking six AS levels and gaining Bs in all of them (6 @ 50). The tariff also considers all such candidates equally, irrespective of the level or number of qualifications or the subjects in which they were taken. In particular, the tariff score includes General Studies A level on an equal footing with all other A levels, whereas many medical schools do not regard it as a full A level. For the present study we have therefore recalculated attainment scores *de novo*, and have considered only A levels, scoring them in the same way as for the YCS, with 10 = A, 8 = B, etc.. For most medical schools it is A levels that matter, and not combinations of A and AS levels. UCAS was unable to provide data on Scottish Highers, and therefore our analysis is restricted to those taking A levels, being typically candidates outside of Scotland.

For convenience and ease of interpretation we have confined our analyses to applicants who were aged under 21 at the time of application (making them broadly equivalent to the YCS data), and who were resident in the UK, giving a total sample of 976,007. Some applicants had not taken A levels, for various reasons, in particular being resident in Scotland or Wales, and taking Highers or Baccalaureate, and we therefore considered only the 531,333 candidates who had results for at least three A level subjects (excluding General Studies). A very small number of candidates, 2642, had five or more A levels, and for practical reasons we excluded them also, leaving 528,691 applicants, 473,781 with three A levels and 54,910 with four A levels. For simplicity, for candidates with four A levels we have considered only the grades from their three best results, meaning that for all candidates the maximum number of points is 30, which is equivalent to AAA. Although this might seem to devalue the additional qualification obtained by those with four A levels, it is worth noting that while 10.5% of the applicants with three A levels had attained the maximum score of 30 points, that score was attained by 48.9% of the applicants with four A levels, showing that those taking four A levels tended to be amongst the very highest achieving of all applicants.

#### A level results of UCAS applicants in relation to the YCS population

**Figure 7:**
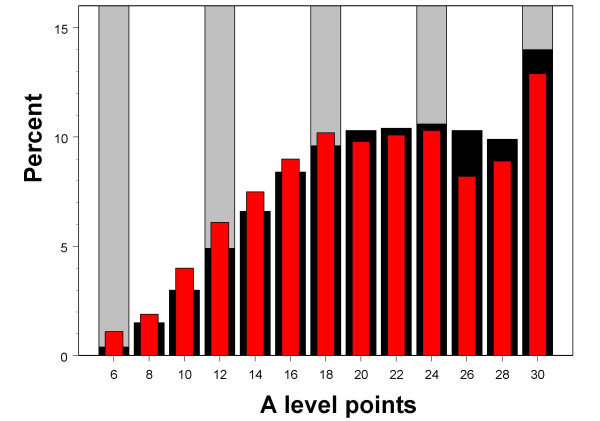
Distribution of points attained on best three A-levels (see text), for students in the YCS (red bars), and for all students applying to UCAS in 2003 (black bars). Grey bars in the background correspond to 6, 12, 18, 24 and 30 points, equating to EEE, DDD, CCC, BBB and AAA.

In order to compare UCAS applicants with all those taking A levels, the data from the YCS A levels were expressed in the same way as for the UCAS applicants, considering only students gaining three or four A levels at grade E or above, and for those taking four A levels, using the three best results. This score correlates highly with the total points score used in the earlier analyses (r = .957). Figure 7 shows the possible scores for three A levels, from 6 (EEE) through to 30 (AAA). The YCS data were compared specifically with the 2003 UCAS applicants, since that cohort was the closest in time of the UCAS cohorts to the YCS cohort. Although the distributions are broadly similar, in particular being > censored= in a similar way at the top end, the mode in both cases being at AAA due to no higher grades being available, it is also clear that the YCS group (red bars) has somewhat more individuals at lower grades, and fewer individuals at the highest grades. UCAS applicants therefore have somewhat higher grades than the population as a whole, which would be expected as only the better A-level candidates will apply to UCAS, but otherwise have a broadly similar A level distribution to that found in YCS. .

#### A level results in UCAS applicants in relation to ethnicity

**Figure 8:**
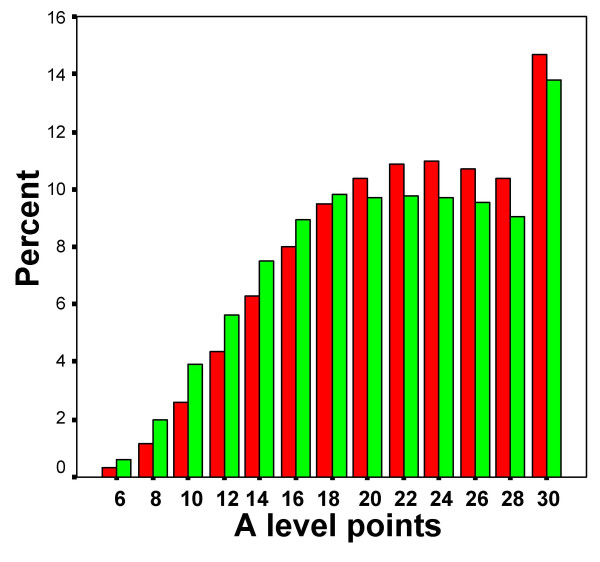
The distribution in all UCAS applicants of A level points gained by W students (red bars) and NW students (green bars).

Figure 8 shows the distribution of A level grades in the UCAS dataset in relation to ethnicity. The broad distributions of each group are similar to those in Figure 7, with a mode at 30 points due to censoring. A simple comparison of W and NW students finds that W students have a higher achievement than NW students (Table 5). Of interest is that the overall effect size comparing W with NW is B 0.140, which is similar to the effect size found in the YCS data of B 0.141 (see Table 4). An achievement of grades ABB or above (i.e. 26 points or equivalent, which can be regarded as a high A-level achievement), is reached by 35.7% of W applicants overall (i.e. for any subject), compared with 32.4% of NW applicants (Chi-squared = 308.8, 1df, p < .001, odds ratio = 1.160, effect size = B.082).

**Table 5:** Comparison of A-level achievement in White and non-White applicants in the UCAS data, for all applicants, applicants taking Chemistry, applicants applying to medical school, and applicants accepted for medical school.

	White Mean A-level scores (SD)	Non-White Mean A-level scores (SD)	Significance	Effect size
All applicants	21.92 (5.93) N = 443,038	21.09 (6.31) N = 74,262	t = 34.397 p < .001	d = -.140
All applicants taking Chemistry	23.83 (6.03) N = 72,925	22.92 (6.35) N = 24,626	t = 20.26, p < .001	d = -.151
All medical school applicants	27.29 (3.56) N = 15,073	26.29 (4.41) N = 8,174	t = 18.81 p < .001	d = -.282
All medical school entrants	28.77 (1.97) N = 9,945	28.54 (2.38) N = 4,373	t = 5.89 p < .001	d = -.114
	White N (%)	Non-White N(%)		
All applicants: percent gaining ABB or higher	158,373/443,038 = 35.7%	24,074/74,262 = 32.4%	χ^2 ^= 308.8 (1) p < .001	OR = .862 (CI .848 – .877) d = -.082
All applicants: proportion taking Chemistry	72,925/443,038 = 16.5%	24,626/74,262 33.2%	χ^2 ^= 11,593 (1) p < .001	OR = 2.52 (CI 2.48 – 2.56) d = .-511
Medical school applicants: percent gaining ABB or higher	11,977/15,074 = 79.5%	5815/8174 = 71.1%	χ^2 ^= 204.3 (1) p < .001	OR = 0.634 (CI .60 – .68) d = -.252
Medical school applicants: percent gaining AAA higher	6,893/15,074 = 45.7%	3098/8174 = 37.9%	χ^2 ^= 132.6 (1) p < .001	OR = .724 (CI .69–.77) d = -.178
Medical school acceptances: percent gaining ABB or higher	9509/9945 = 95.6%	4109/4373 = 94.0%	χ^2 ^= 17.9 (1) p < .001	OR = .714 (CI .61 – .84) d = -.186
Medical school acceptances: percent gaining AAA higher	6083/9945 = 61.2%	2586/4373 = 59.1%	χ^2 ^= 5.24 (1) p = .022	OR = .919 (CI .854–.988) d = -.047

#### Applicants taking A level chemistry

**Figure 9:**
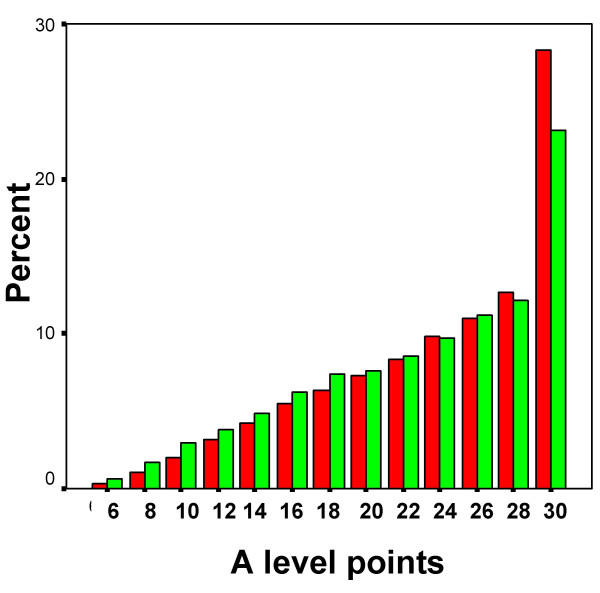
The distribution of A-level points for all UCAS applicants who have taken A-level chemistry, separately for W students (red bars) and NW students (green bars).

Although the UCAS database has 517,300 university applicants with three or more A levels, only 97,551 (18.9%) of these have taken A level chemistry, and hence can be considered as being in the > real pool = of possible entrants to medical school. Amongst those taking chemistry, 25.2% are non-white, compared with 11.8% of those not taking chemistry (odds ratio = 2.518, effect size = .510), again, a very similar figure to the odds ratio found in the YCS data of 2.735. The mean total A level points of NW students taking chemistry is significantly less than that for W students (NW: mean = 22.92, SD = 6.35, N = 24626; W: mean = 23.83, SD = 6.03, N = 72925; t = 20.26, p < .001; effect size = B.151), as can be seen in Figure 9, where it is very clear that students taking A level chemistry are a high-achieving sub-group of students compared with the population in general (Figure 8).

#### Applicants to medical school

**Figure 10:**
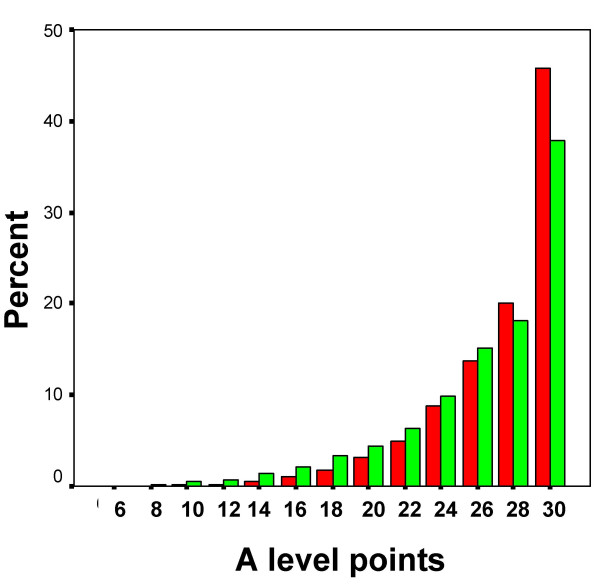
The distribution of A-level points for all medical school applicants, separately for W students (red bars) and NW students (green bars).

For the 23,247 applicants to medical school, of whom 8,174 (35.2%) were NW, the average number of A level points was significantly higher in the W applicants than the NW applicants, with an effect size of B.282 (see Table 5). Figure 10 shows that the W applicants have a higher proportions of 30 points and 28 points, whereas NW applicants have a higher proportions of all other grades. In view of the non-normal distribution, a non-parametric test was used to confirm the difference in distributions (Mann-Whitney U = 54151179, z = 15.99, p < .001). Table 5 also shows that NW applicants were significantly less likely to achieve 26 points or more, which is equivalent to ABB, (odds ratio = 0.634, effect size = B.252), or achieve grades of AAA (odds ratio = .724, effect size = B.178).

#### Entrants to medical school

**Figure 11:**
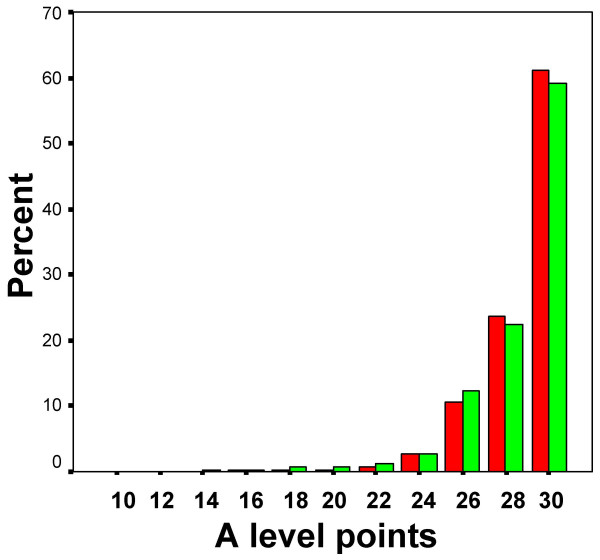
The distribution of A-level points for all medical school entrants, separately for W students (red bars) and NW students (green bars).

Of the 14,318 entrants to medical school, the average number of A level points was 28.77 (SD 1.97) in the 9,945 W entrants, and 28.54 (SD 2.38) in the 4,373 NW entrants, a highly significant difference (t = 5.89, 14316 df, p < .001), with an effect size of B.114. Figure 11 shows the distribution, with W candidates being more likely to have 30 and 28 points, and NW candidates to have 28 points or less. In view of the skewed distribution, a non-parametric test was used to confirm the difference in distributions (Mann-Whitney, U = 21057283, Z = 3.453, p < .001). Table 5 also shows that NW acceptances were significantly less likely than W acceptances to have achieved 26 points or more, equivalent to a grade of ABB or more (odds ratio < .001; odds ratio = .721, effect size = -.186), or to achieve a grade of AAA (odds ratio = .919, effect size = B.047).

### Exploring the censored distribution of A level attainment in W and NW students

The distribution of A level attainment in university applicants in general, shown in Figure 7, is an important one that is capable of further analysis. It can be seen that to a first approximation the distribution is normal, with a mode at about 20 points (i.e equivalent to about BCC) but with a wide distribution around that, and, in particular a clear second mode at the maximum of 30 points (AAA). It should also be noticed that the distribution tails away towards the left-hand end of the distribution, the very lowest points possible with three A levels being 6 (i.e. EEE).

In interpreting this distribution, a clear distinction should be made between distributions that statisticians describe as *censored *and those described as *truncated*. In a clinical survival analysis, patients may be followed up for, say, five years. During that time, some will have died, and their precise survival will be known. Others, however, will have survived for the whole of the five years but will, inevitably, succumb at some future time, but all that can be said of them is that their survival is at least five years; these data are *censored*, the number of observations being known but their precise value being unknown. In contrast, a study may look only at a group of patients whose blood pressure is known to be at least 160 mmHg, and the distribution of blood pressure for those individuals is *truncated*, no individuals being included below the threshold level. The key difference is that for the censored data, all of those surviving for beyond the measured time are included in the final bin of the histogram, whereas in the case of truncation, those not included are not shown anywhere in the histogram.

The A level data of Figures 7 to 11 are truncated at the lower bound, as individuals are only included who have gained a minimum of 6 points from 3 A levels. In contrast, at the upper end of the distribution, individuals who could have gained more than 30 points (AAA) if the marking scheme had allowed it, by, for instance, including hypothetical grades of A*, A** and A***, gaining 12, 14 and 16 points respectively, are forced into the highest bin of the histogram, AAA, because they cannot achieve any higher result given the marking scheme. The A level distribution is therefore censored at the top end (and that accounts for the clear second mode in Figures 7 to 11 at 30 points).

**Figure 12:**
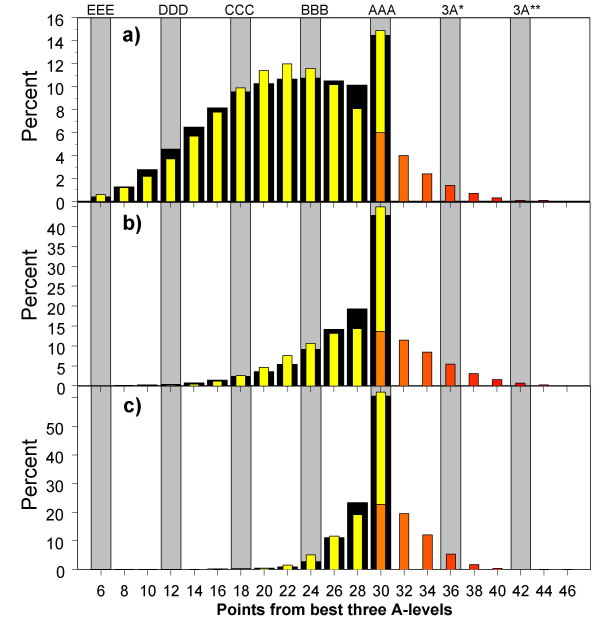
The black bars show the observed distributions of A-level grades attained by a) all UCAS applicants, b) all medical school applicants, and c) all medical school entrants. The yellow bars show the fitted distribution which has been estimated on the basis of a normal distribution which has been truncated at 6 points and censored at 30 points, with all individuals scoring more than 30 points being included in the 30 point bin. The orange bars show the proportions of individuals who would have been expected to gain 32, 34, 36 etc. points, where 36 points can be construed as three A* grades, 42 points three A** grades, etc..

When a distribution is censored, as in Figures 7 to 11, then although a simple mean can be calculated in the traditional way, it makes more sense to consider the mean of the true or latent, underlying, distribution which had generated the censored distribution that was actually found. The latent distributions for all UCAS applicants were calculated separately for W and NW candidates, and the simple censored distribution was fitted (further details are described elsewhere [38]). For medical school applicants and acceptances, the parameters were estimated jointly for both applicants and acceptances, using a two-parameter logistic function to estimate the selection ratio at each level of achievement, with all parameters calculated separately for W and NW applicants. As an example of the fitting process, Figure 12 shows the observed distribution of A level scores for all applicants, all applicants for medical school, and all acceptances for medical school. The wide black bars in Figure 12a show the percentage of all UCAS candidates gaining the various grades. The ceiling at 30 points (AAA) is clearly shown, and is gained by 14.5% of these applicants. The modelled, normal distribution, censored at 30 points, showed a reasonable fit to the data (yellow bars, mean = 22.1, SD = 6.60). The red bars in Figure 1a show the expected distribution of applicants gaining more than 30 points from 3 A levels, assuming similar scaling of the current A levels, and with A* = 12, A** = 14, etc.. Figures 12b and 12c show similar distributions for medical school applicants and acceptances, the true mean (SD) being estimated as 28.3 (5.5) for applicants and 30.1 (4.5) for acceptances.

**Figure 13:**
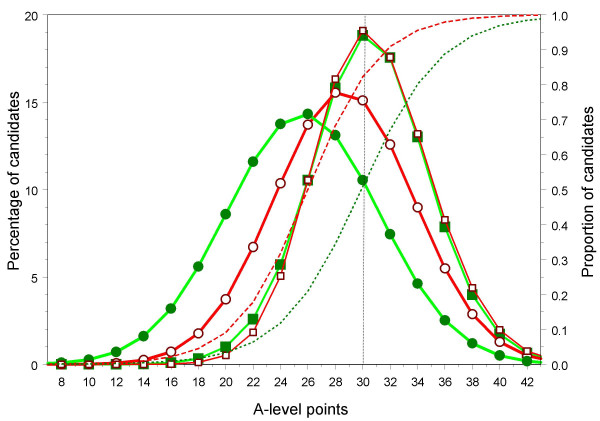
The estimated, underlying, uncensored distributions of A-level ability in medical school applicants and acceptances are shown for W candidates (red lines and open points) and NW candidates (green lines and solid points). The leftmost two lines with circles joined by thick lines show the fitted distribution for the percentages of *applicants *with particular A-level points, whereas the rightmost two solid lines with square points show the fitted distribution for the percentages of *entrants *with particular numbers of A-level points; all four lines use the left-hand ordinate. The dashed, ascending lines use the right-hand ordinate and show the fitted proportions of candidates with particular number of A-level points who are accepted, red for W applicants and green for NW applicants.

Table 6 shows the estimated parameters of the uncensored distributions for W and NW applicants, medical school applicants and medical school acceptances. The first four columns are relatively uncomplicated and show the fitted mean and standard deviation of A-level grades for all university applicants, the NW applicants having a lower mean and slightly higher standard deviation than the W applicants. The reminder of the table is best interpreted in conjunction with Figure 13, which shows the estimated distributions and selection functions for medical school applicants and acceptances. The red and green circles on Figure 13 show the fitted distributions of W and NW medical school applicants respectively, the NW distribution being clearly shifted to the left by about 0.6 standard deviations (the precise parameters are shown in the last three columns of Table 6). Similarly red and green squares show the fitted A-level grade distributions of W and NW medical school acceptances, the parameters being shown at the bottom of Table 6. The selection functions, which show the proportions of applicants who are accepted, in relation to A-level points, are shown as separately for the W and NW applicants as the green and red dashed lines, and the parameters of the function are shown in Table 6.

**Table 6:** Parameter estimates for the latent (uncensored) distributions in all applicants, and in medical school applicants and acceptances, separately for W and NW candidates.

	All applicants			Medical school applicants and acceptances		
	W	NW	Effect size	W	NW	Effect size

Estimated true mean in applicants	22.29	21.45	-.129	28.63	25.63	-.592
Estimated true SD in applicants	6.53	6.92	-	5.06	5.53	-
Selection process: Slope	-	-	-	.225	.200	-
Selection process: Intercept	-	-	-	26.00	29.9	-
Estimated true mean in acceptances	-	-	-	30.80	30.51	-.068
Estimated true SD in acceptances	-	-	-	4.23	4.36	-

The effect size of -.129 for the A level performance of all NW applicants is similar both to the simple, unadjusted, effect size of -.141 found for applicants in the UCAS data (Table 5), and the effect size of -.140 found for A level points in the YCS data (Table 4). In contrast the true effect size of -0.592 for medical school applicants shown in Table 6 is considerably larger than the effect size calculated from the raw data that is shown in Table 5 of -.282, showing that the censoring of the data has distorted the true picture. Similarly, the raw effect size of -.114 for medical school acceptances in Table 5 is larger than the adjusted effect size of -.068 in Table 6. Censoring of the data by the artificial ceiling of AAA in A levels therefore distorts the estimates of effect size.

## Discussion

This paper has analysed two very different databases, in order to answer questions about the academic background and qualifications of medical students from ethnic minorities. The questions it has asked have, inevitably, been simplified, particularly in so far as the studies have looked only at A level grades, in those students who have taken three or four A levels, and they have looked only at a division of the population into just two groups, White and non-White. No doubt far more subtle analyses could carried out, but those presented here are sufficient for a basic analysis of the issue.

The starting point for the paper is that in a range of studies, medical students and doctors from ethnic minorities have underperformed in different types of examination, both undergraduate and postgraduate. Although it is inevitably tempting to attribute underperformance in some examinations, such as clinical assessments, to factors such as direct or indirect discrimination on the part of examiners, perhaps because of cultural insensitivity or other reasons, all such explanations have problems in explaining why candidates from ethnic minorities underperform in assessments of knowledge which use multiple-choice questions and are marked by a computer.

A crucial but implicit assumption of many studies of underperformance by students from ethnic minorities is that W and NW students are initially equivalent in their academic ability. Since selection has taken place on the basis of an academic criterion, then it might seem reasonable to infer that equality of intellectual ability is present in students from different groups. A particularly clear example comes from Esmail, commenting on the original findings in Manchester in 1995 that a higher proportion of NW students were failing their final examinations.

AWe found a significant statistical association between men with Asian names and failing clinical exams. ... These were not substandard students. They were accepted to Manchester University *on the basis of the same A level criteria as everyone else*.@ [7] (our emphasis)

It is a seductive and powerful argument, and one that in some form or another, many of us have used. However, the data in the present analysis suggest it is not correct, and that finding has a number of important implications, particularly in an area where research is quoted in the context of Aracial discrimination≅ [39] and Aracism in medicine≅ [40]. Despite being selected Aon the basis of the same A level criteria as everyone else≅, there are several mechanisms by which NW students may still underperform at medical school.

### i) Different distributions of educational achievement in the W and NW populations

The YCS data are important because they show the achievement of a large, representative population sample on an examination, the GCSE, which is taken by almost all students in the UK population at the age of 16, and for which teachers and schools are rewarded for their students performing as well as possible. There is therefore every reason to believe it is taken very seriously. Figure 1 shows that NW students have both a lower mean attainment and a somewhat greater variance of attainment at GCSE than do W students. The reasons for that are complex, and as Figure 2 shows, it is in large part a reflection of NW students tending to come from lower socio-economic groups, and their parents being less likely to have progressed into post-compulsory education at A level or degree standard, both factors influencing GCSE achievement, factors which are probably still important in the cohort of UK children born in 2000 [41]. Noteworthy, though, in Figure 2, is that ethnicity, despite its clear effect in a simple analysis, has little effect after other variables are taken into account, its effects mainly being mediated via parental variables. It is also important that ethnic minority students are equally likely to attain 6 or more grade As at GCSE, suggesting that performance at the top end of the distribution, at a level which is probably needed to achieve medical school admission, is equivalent in white and non-white students. The overall difference in mean GCSE performance, coupled with a wider variance in non-white students, suggests that differences in socio-economic and parental educational factors are mainly responsible for non-white students underperforming. As more non-white students enter higher education and subsequently have children, that difference is likely to diminish.

The YCS data also show that there is a very clear relationship between performance at GCSE and subsequent performance at A level (Figure 5), and that the structural form of the relationship is very similar in W and NW students, suggesting that GCSEs meet the Cleary test for equivalent predictive validity [42] B although see Chung-Yan and Cronshaw for criticisms of the Cleary test [43]. Good GCSE results therefore should predict good A level results in a similar way in both W and NW students. Although the simple correlation between GCSEs and A levels is strong, with a correlation of about 0.63, it is inevitably an underestimate of the true correlation, since measurement error of the two examinations has not been taken into account, and neither has the effect of restriction of range (A levels only being taken by those with better GCSE results). The true structural (disattenuated) correlation is likely to be of the order of 0.7 to 0.8, or so.

Because NW students do less well at GCSE, it is therefore to be expected that on average they are also likely to do less well at A level, solely on that basis.

### ii) Choice of GCSEs, choice of A levels and inappropriate aspirations

Although the taking of GCSEs is, in effect, compulsory, the particular subjects chosen to be taken, as well as their number, is in part a matter of choice in a decision made jointly by students, their schools, and their parents. Some subjects are undoubtedly easier than others [35], and some students choose, for whatever reason, to take harder subjects, a decision, as shown in Figure 2, which is influenced by parental education and occupation, and by schools. At GCSE, there is evidence that students choosing to take harder subjects in fact perform *better*, probably because these students are already higher achievers and schools, parents, or they themselves, wish them to be stretched.

Similar considerations apply at A level, although the choice of A level subjects, and indeed the decision to take A levels at all, are both made in the light of GCSE grades already attained. Of particular importance are the results shown in Figures 3 and 4. It is clear that, for any particular level of GCSE attainment, NW students are much more likely to take A levels in general, and science and chemistry A levels in particular. Since GCSEs are seen to be powerful predictors of A level attainment, the implication is that NW students will, on average underperform yet further at A level than would be predicted on the basis of their relatively poorer overall performance at GCSE. That can be seen in the YCS data, where the mean A level attainment of NW students is significantly less than that in W students. Inappropriate aspirations are partly involved, as seen in the significantly higher proportion of NW students who intend to take A levels but in fact do not do so, those non-takers also having lower GCSE attainment than those who do go on to take A levels. Connor et al [21] have noted that NW students were more likely to cite parental influence in their educational choices, and that Asian parents aspire to their children having Aprofessional≅ careers, such as in medicine or law, and that this may in part explain the increased proportion of Asians studying medicine.

### iii). Poorer NW A level attainment in the pool of UCAS applicants

The majority of applicants with reasonable A levels currently apply to university (as can be inferred from Figure 7), and there are other similarities between the YCS and UCAS data, which suggest that the data sets are equivalent in many ways. In particular, it is important to note that in the YCS data, and using measures of effect size, the NW students performed 0.141 SDs below the W students, and in the UCAS data the NW students performed 0.140 SDs below the white students (see Figure 7), showing the datasets are very similar in the effects they are finding, with the advantage that the UCAS dataset is extremely large, and has information on the small minority of students who apply to and are accepted by medical school.

### iv). The A level achievement of applicants and acceptances at medical school

Figures 9 and 10 make very clear that medical school applicants, and particularly medical school acceptances, are a very highly selected academic elite from amongst the entire pool of those applying to university, the modal A level attainment being at the maximum possible score of three As at A level. Despite that, it is also clear, a) that medical acceptances have higher attainment than those not accepted, and b) that both in applicants and in acceptances, the NW candidates have significantly lower levels of achievement than the W candidates. The mechanisms for that in applicants cannot be explored directly, but it is likely that differential aspirations are involved. Likewise, the reasons for the difference in those accepted cannot be analysed further, but it might, for instance, be argued that extenuating circumstances are more likely to be important in NW students than W students, and hence they are admitted with somewhat lower achievement than W students. However, that explanation is not compatible with the clear finding, in the selection functions shown in Figure 13b, that selection for non-white applicants is *stricter *than for white applicants, the 50% point of the logistic function being at 26 points for W applicants and 30 points for NW applicants. Similar differences have been reported previously, in a study of selection for UK medical schools in 1996 and 1997 [44], the likelihood of a medical school offer being lower for NW applicants at all levels of A level achievement.

It is harder for NW applicants to enter medical school, even when they achieve equivalent A level grades to W applicants, although the net result of selection is that the A level distributions of White and non-white entrants are much more similar than in applicants. Nevertheless, W acceptances still have significantly higher A level grades than do NW, although the effect size is relatively small (but see below for further discussion).

### v) Selection from groups of different mean levels of ability

The mean A level achievement of NW university applicants and entrants is lower than that of W students, for whatever reason (see Figures 7, 8, 9, 10, 11, 12, 13). That fact alone means that in a fair selection system in which all individuals of whatever group who are over some critical threshold are accepted, the mean achievement of those selected from the group with the lower average performance, will also be significantly lower than in the higher achieving group. As an example, consider the entire distribution of candidates in Figure 9 who have taken A level Chemistry and hence can be considered as in the > real pool = of potential medical students. Simply select all of those who have 26 or more points at A level i.e. grades of ABB or better. Altogether, 25.0% of those taking A level chemistry are NW (compared with the 14.4% of UCAS applicants overall who are NW). However, only 23.0% of those meeting the 26 point criterion are NW, compared with 27.0% of those in the W group, an odds ratio of 1.235 (equivalent to an effect size of -.117). More crucially, amongst those selected on the 26-point criterion, and despite the extreme narrowness of the range of A levels selected (only 26, 28 or 30 points), the mean score of the W students (28.67, SD = 1.61) is higher than the mean score of the NW students (28.53, SD = 1.64), a highly significant difference (t = 8.390, p < .001, effect size = -0.090). Of particular interest is that 54.6% of the 37752 W students had the maximum 30 points, whereas only 50.1% of the 11294 NW students had 30 points (p < .001; odds ratio = 1.198, effect size = -0.100). To some extent that difference in entry qualifications is likely to perpetuate itself as students continue through their education, particularly given that A levels have been shown to be continuing predictors of undergraduate and postgraduate medical school performance [28,29].

#### Effect sizes

Throughout this paper we have presented differences in attainment of white and non-white students in terms of standard effect sizes, with *d *being the difference between the groups in terms of standard deviations of the white students (the larger group). Perhaps the most interesting result concerns acceptances at medical school, where, in Tables 5 and 6, effect sizes have been calculated in four different ways, giving values of B.186, B.114, B.068 and B.047, with a mean of -.10, or one tenth of a standard deviation, which can be generally regarded as a small effect (although it is much larger than some of the very small effects found in therapeutically important clinical trials of new drugs [45]). The effect is a little smaller, albeit broadly similar, to the overall effects found at GCSE (B.151) and A level (B.141) in the YCS, and at A level in the UCAS data for all applicants (B.140), for applicants taking chemistry (B.151), although it is substantially lower than in medical school applicants (B.282). There seems therefore no doubt that the under-attainment of NW students applying to university, be it for medicine or in general, is part of a broader pattern of under-attainment throughout pre-school education, compulsory and post-compulsory education, perhaps exaggerated at A level by greater educational aspirations in NW students. However, and it is an important > however =, the effect found in medical school acceptances of about B.10 is substantially smaller than the effect found in undergraduate and postgraduate medical examinations of about B.32. It seems unlikely, therefore, that the earlier underachievement of NW students at GCSE and A level alone can entirely explain the poorer performance of NW students at doctors at medical school and beyond. Nevertheless, the difference is still likely to explain some of the effects found. If NW students come from a normal distribution with an effect size of -.10 relative to the W students, then it can be expected that amongst those performing 1 SD below the white mean there will be 17% more NW students, and amongst those 2SDs below the white mean, there will be 27% more NW students (so that if about 5% of W students were failing, then about 6.2% of NW students would be likely to fail, a 23% excess, with about 5 NW failures for every 4 W failures).

#### A levels and selection

There seems little doubt that A levels are still the major component of selection in most medical schools, with typical requirements being AAA, AAB or occasionally ABB [46]. Most schools however also say that suitable personality, motivation, experience and other factors are also necessary. Although A levels are clearly important, it can be seen in Figure 13 that success is not guaranteed, even for white applicants with grades of AAA (of whom only 88% were accepted in the UCAS data). Although it is tempting to believe that this reflects medical schools using other criteria for selection, that conclusion is far from secure. In particular it should be remembered that most applicants to medical school are applying pre-A level, and hence offers and rejections are made not on A levels attained, but on GCSEs attained, and teachers = estimates of A levels that are likely to be gained. If a medical school believes, for whatever reason, that a candidate will not attain their likely offer (say, AAB), then they may well reject a candidate, particularly since it is a particular problem for a school to have more rather than less candidates achieving their offers, schools being legally bound to accept all applicants who meet their entry requirements but also having strict government quotas on the numbers of medical students who can be accepted. Subsequently such candidates may well achieve grades of AAA but in the absence of an offer they cannot at that time gain a place (although they may gain one in the next year). The result, even in the absence of selection on the basis of personality, or whatever, would be that not all AAA candidates would be accepted. The UCAS data do not allow these possibilities to be distinguished.

#### The implications of differences in educational attainment of ethnic minority students

Although NW students have been selected on similar criteria to W students, they not only have lower educational attainment than W students, but the population from which they are drawn, the pool of all NW medical school applicants, has lower educational attainment than those in the pool of all W medical school applicants. As a result, and using the estimated parameters of the censored normal distributions provided in table 6, while the mean score of W acceptances is 0.43 standard deviations from the mean of W medical school applicants, the mean score of W acceptances is 0.88 standard deviations above the mean of NW medical school applicants. In such a situation there is likely to be differential regression to the mean, with NW applicants performing less well than W applicants.

#### Other factors contributing to underperformance of NW students and doctors

Although this study has identified a small but real effect of educational background which is likely to mean that some NW medical students and doctors underperform in examinations, it clearly cannot account for the entire effect found in earlier studies. What other factors also account for the underperformance is still very unclear. NW students may have somewhat different motivations [47], but it is not clear as yet if these differences predict outcome. Differences in learning style and a range of other factors have been looked at previously [9], and typically any differences do not account for differences in W and NW examination performance. Further studies are needed of this phenomenon, which provides a serious challenge to the discipline of medical education.

## Conclusion

Non-white medical students have somewhat lower A level grades than white medical students, the effect size being about -0.10 standard deviations, which is similar, if a little smaller, than the relative underperformance of non-white students at GCSE and A level. Although the effect is reliable, it is substantially smaller than the relatively large effect size found in undergraduate and postgraduate medical education, of about -0.32 standard deviations, although it probably contributes something to that effect.

## List of abbreviations used

GCSE: General Certificate of Secondary Education; NW: Non-White; UCAS: Universities and Colleges Admissions Service; W: White; YCS: Youth Cohort Study

## Competing interests

The author(s) declare that they have no competing interests.

## Authors' contributions

ICM and KW had the original idea for the study. JD provided finance for obtaining the UCAS data. ICM was primarily responsible for the data analysis. ICM, KW and JD were all involved in the interpretation of the results, and contributed to the drafting of the manuscript.

## Pre-publication history

The pre-publication history for this paper can be accessed here:



## Supplementary Material

Additional file 1 Ethnicity breakdown in YCS and UCAS data. Click here for file
