# Supplementary material for: The educational background and qualifications of UK medical students from ethnic minorities
Source: BMC Med Educ. 2008 Apr 16;8:21. doi: 10.1186/1472-6920-8-21 (PMC2359745; doi:10.1186/1472-6920-8-21)
Supplement: Additional file 1 — Ethnicity breakdown in YCS and UCAS data. [file 1472-6920-8-21-S1.pdf]

Supplementary File

Supplementary table 1: Ethnicity breakdown in YCS and UCAS data.

		YCS - Sweep 1	UCAS: All applicants, 2003-5	UCAS: UK nationals aged under 21 with 3 or 4 A-levels, 2003-5	UCAS: UK medical school applicants aged under 21 with 3 or 4 A-levels, 2003-5	UCAS: UK medical school entrants aged under 21 with 3 or 4 A-levels, 2003-5
**White (including English, Irish, Scottish, Welsh, British, etc)**		**11273 (86.4%)**	**965647 (65.0%)**	**443038 (83.8%)**	**14875 (63.7%)**	**9747 (68.5%)**
**Black - total**		**313 (2.4%)**	**58790 (4.0%)**	**10464 (2.0%)**	**861 (3.7%)**	**341 (2.4%)**
	Caribbean	120 (0.9%)	16104 (1.1%)	3291 (0.6%)	89 (0.4%)	33 (0.2%)
	African	120 (0.9%)	38634 (2.6%)	6416 (1.2%)	721 (3.1%)	287 (2.0%)
	Black - other	72 (0.6%)	4052 (0.3%)	757 (0.2%)	51 (0.2%)	21 (0.2%)
**Asian - total**		**992 (7.6%)**	**120472 (8.1%)**	**41700 (7.9%)**	**5414 (23.2%)**	**2925 (20.5%)**
	Indian	355 (2.7%)	49883 (3.4%)	22886 (4.3%)	2504 (11.7%)	1485 (10.4%)
	Pakistani	329 (2.5%)	33462 (2.3%)	9846 (1.9%)	1411 (6.0%)	642 (4.5%)
	Bangladeshi	128 (1.0%)	10494 (0.7%)	3433 (0.6%)	345 (1.5%)	158 (1.1%)
	Chinese	72 (0.6%)	4069 (0.3%)	1917 (0.4%)	381 (1.6%)	120 (0.8%)
	Asian - Other	108 (0.8%)	14636 (1.0%)	5535 (1.0%)	1154 (4.9%)	640 (4.5%)
**Mixed Race**		**127 (1.0%)**	**29196 (2.0%)**	**12743 (2.4%)**	**790 (3.4%)**	**452 (3.2%)**
**Other**		**29 (0.2%)**	**11228 (0.8%)**	**3723 (0.7%)**	**467 (2.0%)**	**239 (1.7%)**
**Not answered**		**315 (2.4%)**	**82599 (5.6%)**	**11391 (2.2%)**	**375 (1.6%)**	**186 (1.3%)**
**Not answered (Overseas) – UCAS only**		**-**	**216718 (14.6%)**	**-**	**-**	**-**
**Total**		**13049 (100.0%)**	**1484650 (100%)**	**528691 (100.0%)**	**23356 (100.0%)**	**14238 (100.0%)**
